# Critical Success Factors and Traceability Technologies for Establishing a Safe Pharmaceutical Supply Chain

**DOI:** 10.3390/mps4040085

**Published:** 2021-11-22

**Authors:** Mona Haji, Laoucine Kerbache, K. M. Mahaboob Sheriff, Tareq Al-Ansari

**Affiliations:** 1 Division of Engineering Management and Decision Sciences, College of Science and Engineering, Hamad Bin Khalifa University, Doha 34110, Qatar; lakerbache@hbku.edu.qa (L.K.); dr.mahaboobsheriff@gmail.com (K.M.M.S.); talansari@hbku.edu.qa (T.A.-A.); 2 Information Systems and Operations Management, HEC Paris, 78351 Jouy-en-Josas, France; 3 Division of Sustainable Development, College of Science and Engineering, Hamad Bin Khalifa University, Doha 34110, Qatar

**Keywords:** pharmaceutical supply chain, traceability technologies, critical success factors, barriers to a safe pharmaceutical supply chain

## Abstract

Drug counterfeits have been an international issue for almost two decades, and the latest statistics show that fake medications will continue to penetrate legitimate pharmaceutical supply chains (PSCs). Therefore, identifying the issues faced by PSCs is essential to combat the counterfeit drug problem, which will require the implementation of technologies in various phases of the PSC to gain better visibility. In this regard, a literature review was conducted to fulfill the following objectives: (i) review the application of traceability technologies in various PSC phases to detect counterfeits; (ii) analyze the various barriers affecting the establishment of a safe PSC and the critical success factors used to overcome those barriers; and (iii) develop a conceptual framework and guidelines to demonstrate the influence of traceability technologies and success factors on overcoming the various barriers in different phases of the PSC. The major finding of this review was that traceability technologies and the critical success factors have a significant influence on overcoming the barriers to establishing a safe PSC.

## 1. Introduction

Various management activities, such as sourcing, conventions, manufacturing operations, marketing, product design, finance, procurement, and all the activities involving logistics, are involved in the development of supply chain management. The wide range of activities sometimes results in flawed business processes for pharmaceutical firms, with issues involving supplier selection, purchasing, warehousing, data accuracy, and the increased complexity and distribution of operations due to their complicated settings. In recent years, pharmaceutical companies have faced challenges related to the development and sale of counterfeit drugs, which are significantly affecting the industry. Counterfeit drugs are fake medicines that resemble authentic medicines, and which are harmful to the health of a patient. The ineffective management of supply chains by manufacturers and distributors has allowed counterfeiters and smugglers to evolve rapidly and become very creative, and excel in producing counterfeit medicines and inserting them into the legitimate supply chains, meaning that many fake medicines go undetected [1]. To counter this issue, global organizations, such as the World Health Organization (WHO), the European Union (EU), and the US Food and Drug Administration (FDA), work together to reduce the spread of this frightening crime to protect the safety and security of patients [2].

Pharmaceutical supply chains (PSCs) are more complex than other types of supply chains. The main reason is the continuous change in the ownership of medications between their leaving the suppliers of raw materials and reaching patients’ hands. The absence of an efficacious supply from manufacturers and distributors poses threats for firms. For instance, errors in the manufacturing processes, medical compounds, storage, temperatures, and distribution tend to make the drugs ineffective and produce adverse side effects or even death. Counterfeit drugs might have no active ingredients, or they might have some harmful ingredients, which are toxic when they are administered to a human being. Such failures of the pharmaceutical firms mean that the consumers pay for products with poor or no medicinal value, which creates many other issues [3]. Including counterfeit medicines alongside real ones often delays the treatment period, causes severe side effects, increases the spread of diseases, and can lead to death [4]. Due to the increasing intensity of the problem over the years, and with increased competition, the pharmaceutical industry has adopted methods for countering the issue. As a result, certain potential remedies have been adopted in the pharmaceutical sector, such as implementing strict regulations, monitoring cold-chain shipping, implementing price controls for prescription drugs, facilitating supply chain visibility, and providing a range of serialized mandates [5].

Given the importance of implementing innovative technologies for overcoming supply chain issues in the pharmaceutical industry, this paper systematically and comprehensively reviews the literature on traceability technologies for detecting counterfeit medicines and ensuring a safe PSC. It assesses the application of traceability technologies, barriers to their implementation, and the critical success factors in various phases of the PSC for achieving a safe PSC and detecting counterfeits. This assessment has significance for the industry with regards to traceability technology and the need to develop an adequate supply chain system.

### 1.1. The State of the Literature

Yu et al. [6] discussed the issues related to China’s pharmaceutical market and focused on implementing radio frequency identification (RFID) technologies to enhance the healthcare system, listing associated barriers. Coustasse et al. [7] also reviewed the wide range of benefits and barriers to adopting RFID technologies and their potential impacts on the hospital supply chain.

Jaberidoost et al. [8] identified the risks facing the PSC and classified them into seven main categories from the perspective of manufacturing companies. Kovacs et al. [9] identified 42 different technologies based on how they are utilized by low- and middle-income countries (LMIC) to analyze and detect counterfeited drugs. Ab Talib et al. [10] focused on reviewing the critical success factors in the context of supply chain management (SCM) and identified the “vital few” and the “useful many” by using Pareto analysis. Hamilton et al. [11] showcased the technologies being used to rapidly identify and detect fake medicines, including Sproxil’s Mobile Authentication Service (MAS), which allows consumers to verify their medications’ authenticity at the point of purchase.

Fadlallah et al. [12] discussed and identified the strategies and critical success factors for effectively fighting counterfeit drugs. Sheikhi et al. [13] identified the key success factor used to improve the efficiency and effectiveness of the drug supply chain, and the study summarized that the technology selected, level of information shared, and average inventory were the main factors that affected overall performance. Mackey and Nayyar [14] explored the existing and emergent digital technologies that can be used to trace high-value medications and validate their authenticity. Ding [15] identified the barriers to a range of potential sustainability measures in PSCs, such as high cost, length of time, ineffective collaboration and coordination, lack of expertise, and unknowledgeable patients.

De Lima et al. [16] reviewed the anti-counterfeit measures for medicines in the PSC and classified them into four main areas: inter-organizational processes and policies, intra-organizational processes and policies, stakeholders’ behavior, and technological implementation. Silva and Mattos [17] identified the critical success factors for an effective PSC and prioritized them using the analytic hierarchy process (AHP) to achieve effective drug traceability. Bottoni and Caroli [18] and Bolla et al. [19] discussed the technologies used to check the chemical characteristics of drugs and detect counterfeits in the active pharmaceutical ingredients (APIs).

Most published review papers have focused on identifying the type of technologies that are useful for detecting counterfeit drugs for a particular phase of the PSC. Nevertheless, other papers have explored factors other than technological implementation which either contribute to a safe PSC or affect adaption. The review of previous literature indicated that traceability technologies, success factors, and barriers were not considered together, and their influences on each other and the phases of the PSC have not been analyzed.

### 1.2. Review Objectives

**Figure 1:**
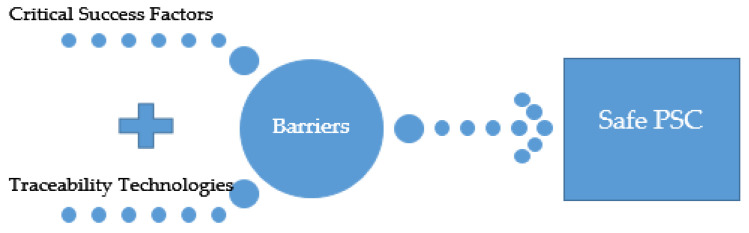
Objectives framework.

This review study integrates and discusses the links between traceability technologies, critical success factors for overcoming barriers, and the detection and solution of counterfeit issues, all of which can contribute to a safe PSC, as illustrated in Figure 1.

This research aims to meet the following objectives:

(i) Review the application of traceability technologies in various PSC phases to detect and solve counterfeit issues;
(ii) Analyze the various barriers affecting the establishment of a safe PSC and the critical success factors for overcoming these barriers; and
(iii) Develop a conceptual framework and guidelines to demonstrate the influence of traceability technologies and success factors on overcoming the various barriers in different phases of the PSC.

The study answers these questions through a literature review that is focused on assessing the key issues related to the development of a safe PSC and the potential problems of the process, identifying where these problems can be solved using traceability technologies.

## 2. Materials and Framework

### 2.1. Review Methodology

**Figure 2:**
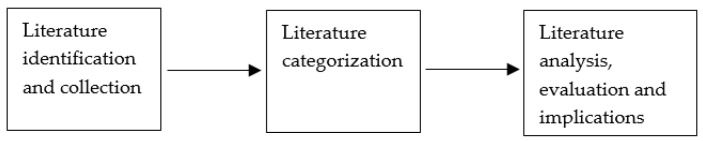
Review methodology.

The review methodology that we have adopted is similar to that used by Yao et al. [20], which has been modified to accommodate the purpose of this paper. The review was divided into three phases, which included the planning of a review in the form of literature identification and collection, conducting the review in the form of literature categorization, and finally reporting the review in the form of literature analysis, evaluation, and implications, as shown in Figure 2, and explained as follows.

#### 2.1.1. Literature Identification and Collection

**Figure 3:**
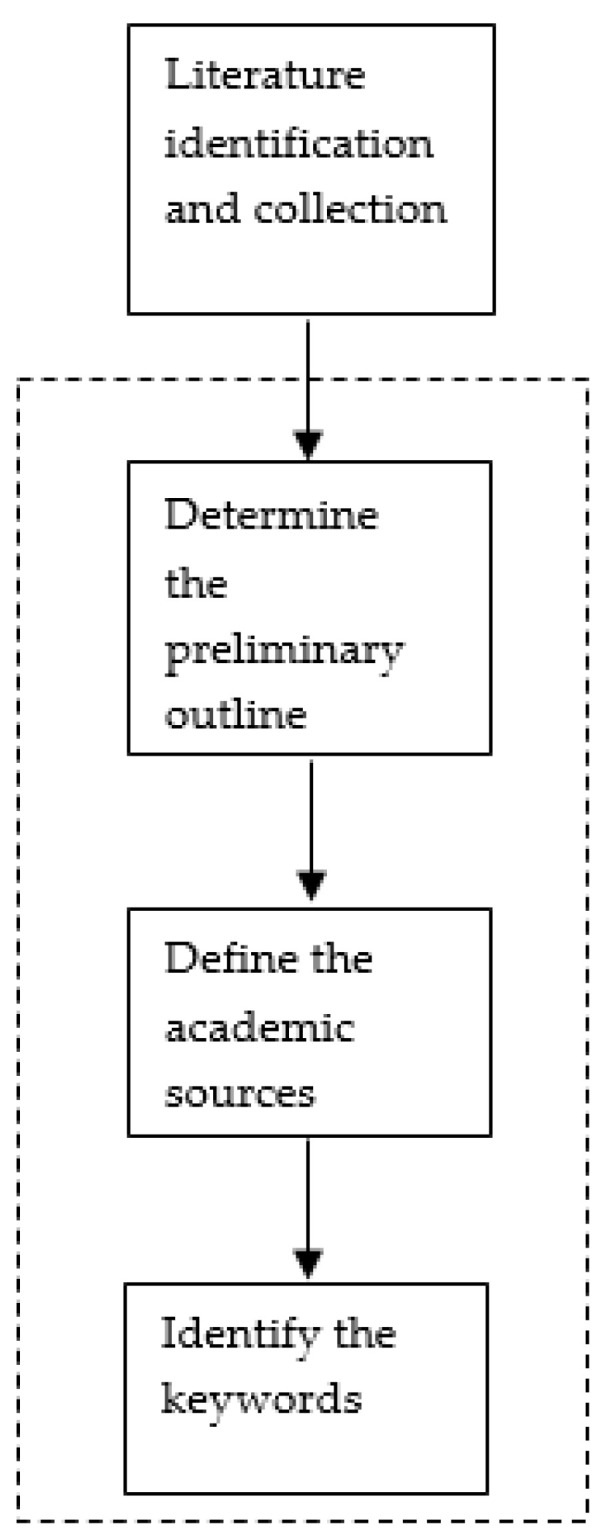
Literature identification and collection.

The literature identification and collection phase comprises three steps, as illustrated in Figure 3.

Step 1: Define the preliminary outline

This paper’s outline was created by defining the phases of a PSC, the main elements of the review framework, and the areas to be discussed regarding the development of a safe PSC. The PSC phases were defined through the review of various sources and articles, drawing on the recurrent concepts and phases of the PSC in different research papers.

**Figure 4:**
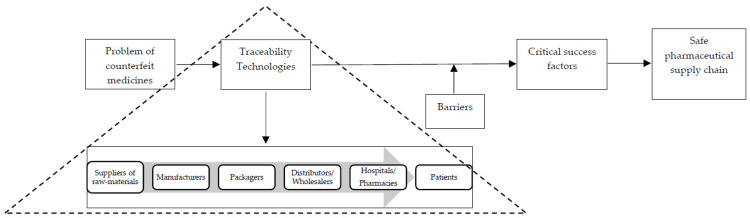
Review framework.

The review framework was developed through the identification of the counterfeit issues faced by PSCs, as shown in Figure 4. Then, we have suggested where the implementation of traceability technologies might occur across the different phases of the PSC, with a definition of the various critical success factors necessary for overcoming the barriers to achieving a safe PSC.

Step 2: Define the academic sources

The literature search was conducted with the help of library search engines, such as “Scopus”, “EBSCOhost”, “Science Direct”, “Emerald Insight”, “Springer Link”, “IEEE”, “Medline”, “Researchgate”, and “Google Scholar”. Other sources were also used to add more value to the review and strengthen the search results, such as Taylor & Francis Online, book chapters, and industrial reports.

Rather than utilize a single academic source, the authors drew on various platforms, including Springer Link, IEEE, and Medlin, which aided the collection of research articles and books that discuss the subjects and relevant knowledge in an elaborated manner. Using all of these search engines ensured a diversified knowledge base that included a range of relevant concepts, and which strengthened the recognition of critical success factors and traceability technologies necessary for establishing a safe PSC. All of the aforementioned databases link to renowned journals and a variety of publications that are relevant to the usage of traceability technologies and other related safety techniques and measures used in the PSC. With the help of appropriate keywords, the most crucial papers were found in each journal and included in this review.

The data related to the subjects were collected in accordance with the research objectives. Relevant data were obtained subject to our quality standards, and we were able to collect an ample amount of relevant material that met the needs of the research, and which ensured the delivery of in-depth, accurate findings. This approach was adopted because it ensured that we were able to pay attention to and collect information appropriate for our collective interpretation.

Step 3: Identify the keywords

In our search, we focused only on papers and secondary sources that were published in English. A range of different keywords for the review were identified that could cover the objectives.

We devised our keywords based on the topic at hand. Specifically, we utilized keywords that would help us develop a comprehensive and elaborate approach for collecting data, and which would therefore include all of the relevant elements that were identified in the review framework according to the subject. Table 1 details the methodology used to identify and search for the keywords.

The keywords were categorized into two areas. The first area looked into the traceability technologies that have been implemented in PSCs to detect counterfeit drugs, while the other focused on barriers and critical success factors for detecting counterfeit drugs and ensuring a safe PSC. Different search strings were used with different combinations of keywords, such as “Traceability technologies AND Drug counterfeiting detection AND Pharmaceutical supply chain”, “Barriers (OR risks) AND Drug counterfeiting detection AND Pharmaceutical supply chain”, and “Critical success factors (OR success factors) AND Drug counterfeiting detection AND Pharmaceutical supply chain”. The words “pharmaceuticals”, “medicines”, and “drugs” have the same meaning and were used interchangeably to refine the search results. The study dealt with various traceability technologies, and the focus was on the latest studies over a period from 2010 to 2021.

#### 2.1.2. Literature Categorization

**Figure 5:**
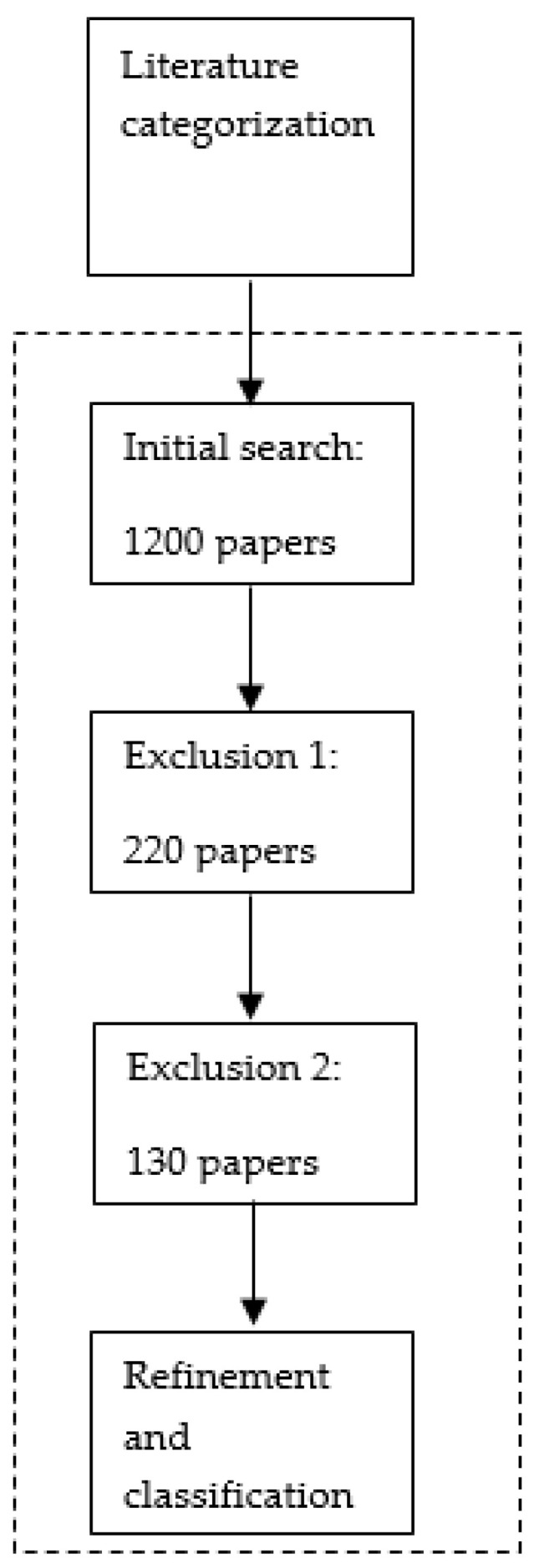
Literature categorization.

Figure 5 shows the sub-steps that were used for categorizing the literature. Initially, the abstract, introduction, conclusion, and the year of publication for each collected research article were reviewed. The articles with no relevance to any of the review objectives were excluded. This literature search led to the collection of 1200 research articles, and each article was classified based on the different topics relating to the predetermined keywords and the sections of the review framework. Then, the content of the different research articles that had been gathered from different sources was compared, and duplicate ones were excluded. The outcome of this step was 220 research articles. Selected research articles were sorted based on their relation the predetermined phases of the PSC and read carefully to ascertain whether or not they were properly specialized in topics relating to the forward supply chain. All of the reverse pharmaceutical supply chain articles were excluded. The outcome of this filter was the shortlisting of 130 research articles for the final review.

#### 2.1.3. Literature Analysis, Evaluation, and Implications

**Figure 6:**
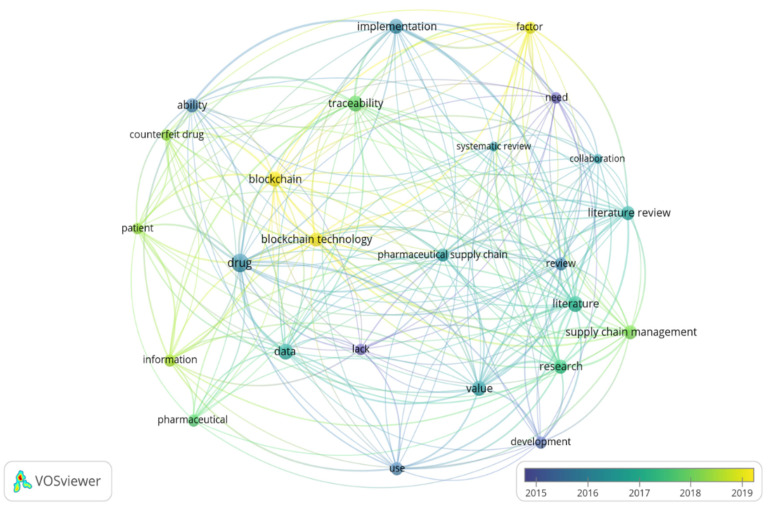
Overlay visualization map.

**Figure 7:**
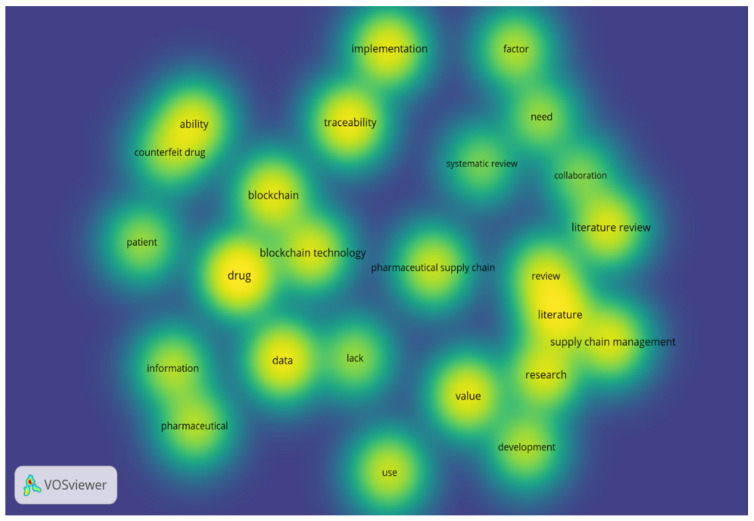
Density visualization map.

The shortlisted papers were exported in Research Information Systems (RIS) format to the VOSviewer software for analysis. The overlay visualization map, shown in Figure 6, details the topics that the articles discussed, such as “Blockchain technology”, “Traceability”, and “Drug counterfeiting”, which started receiving attention in late 2017. The density visualization map in Figure 7 highlights the frequency of focus received by various topics.

## 3. Pharmaceutical Supply Chains

The PSC is the chain responsible for transporting authentic APIs to the manufacturers for production purposes, and for delivering the finished drugs to the patients. Thus, a satisfactory PSC can provide the required quantity of medication at the required time, meet safety and quality standards, and be affordable to as many patients as possible. This section reviews the issues relating to the presence of counterfeit drugs in legitimate PSCs, the application of traceability technologies in different phases of the PSC, barriers, and critical success factors for establishing a safe PSC.

### 3.1. Issues of Counterfeit Medicines in the Legitimate PSC

The WHO has defined counterfeit medicines as “Products deliberately and fraudulently produced and mislabelled concerning identity and source to make it appear to be a genuine product” [21]. Counterfeit medicines are those that not only lack essential ingredients, but which can also contain substandard active ingredients or completely fake ingredients [22].

Counterfeit drugs can appear anywhere in the PSC, where these medications are usually duplicated and sold in the underground economy. The rate of counterfeit drug production has increased due to the disruption that COVID-19 has caused to all supply chains, a lack of business resilience, and the rapid development of technologies [23]. The existence of counterfeit drugs in the legitimate PSC violates the trademark and intellectual property rights of the manufacturers of the original brands and leads to a loss of sales. The brand loses trust from potential customers, and value and profits are adversely affected. Most counterfeit drugs enter the market during peak seasons when the demand for a product is very high. Counterfeited drugs often are the most expensive drugs, such as chemotherapeutic drugs, vaccines, antibiotics, and diabetes drugs. Deceived customers who use counterfeit drugs are those who use the drugs inappropriately or illegally, or who are searching for cheap products, thinking that they are being offered the original at a lower price.

The WHO began its campaign against fake medications in 1980 after the products of drug manufacturers were discovered to have been counterfeited and sold in gray markets [24]. It is estimated that one in 10 medicines sold in the legal markets, particularly in medium- to low-income or developing countries, are falsified [25]. A report provided by US pharmaceutical companies recently stated that their yearly business losses due to counterfeit drugs in the legal PSC were around USD 200 billion [26].

**Figure 8:**
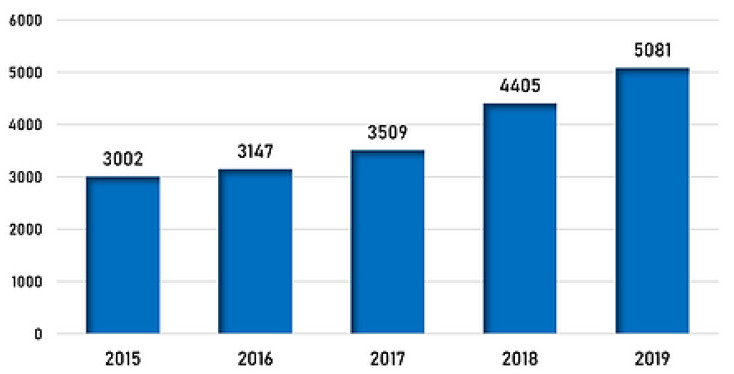
Number of Incidents, CY 2015–CY 2019.

Figure 8 shows statistics provided by the Pharmaceutical Security Institute (PSI) for 2015 to 2019. More than 5000 cases of pharmaceutical crime incidents happed in 2019, with a 15 per cent increase compared to the previous year. The PSI states that the presence of counterfeits increased by 69 per cent over a total of five years [27].

### 3.2. Traceability Technologies in Various Phases of the PSC

#### 3.2.1. Definition of Traceability

The International Organization for Standardization (ISO) defines traceability as “the ability to follow the movement of products through specified stage(s) of production, processing, and distribution” [28]. Bansal et al. [4] indicated that traceability technologies provide unique identity codes that are assigned to each drug and allow for the safe transition through all supply chain stages. These identity codes are provided at an early stage of the supply chain, and the tagged medicine must retain its identity until it reaches the final stage of consumption.

#### 3.2.2. Phases and Technologies

The pharmaceutical market is an attractive place for adulteration and counterfeit crimes due to the high profit gained from illegal trafficking [29]. In his study, Theyel [30] discussed the importance of tracing drugs through all of the stages of the PSC, from the raw materials stage to the consumption-by-patients stage. Drug counterfeiting can occur at any phase of the PSC. However, technologies, such as traceability, can give pharmaceutical firms better supply chain resilience [31] and assist all stakeholders by providing item-level tracking mechanisms. Other research in this area has shown that traceable methods, such as the use of RFID, electronic product codes (EPCs), and business-to-business (B2B) strategies, can ensure that the items are traceable on an individual level, meaning that firms are able to combat the issue of counterfeit medicines [32].

Many different traceability technologies exist in various sizes, shapes, prices, and complexity levels. The earlier the detection of counterfeited medications, the fewer fatal effects the consumer base will experience [33]. The current study overviews the main applications of traceability technologies reported in the literature as follows.

Phase 1: Suppliers of raw materials

The ability of traceability technologies to verify drug authenticity at the production or supply stage is rarely considered because the focus is on technologies that can check the chemical compounds of the medications.

*Blockchain*

There are no qualms about using advanced technology to maintain their operational efficiently, but firms need their systems infrastructure to be robust enough before adopting any new technology [34]. One advanced emergent technology is blockchain. Different technologies have different purposes. Blockchain is more suitable for sharing secured information. Put simply, a blockchain is a time-stamped series of fixed and unalterable data that is recorded and managed by a group of decentralized computers, and which is not owned by any single entity [35]. Blockchain technology was most famously used in Bitcoin and electronic transactions until 2014, when professionals started to discover other useful applications for blockchain [35]. Although blockchain technology has already been proven to be useful in healthcare applications, it is necessary to understand blockchain technology before dealing with its applications [36]. A blockchain is useful for sharing information in a manner that makes it challenging to adulterate the information [37]. This technological aspect can help overcome the vulnerabilities and challenges of various systems, including the safety, consistency, and accessibility of information.

The structure of blockchain technology has been designed so that it is difficult to make changes in the information without being noticed because all stakeholders and organizations in a supply chain share this information [38]. Hence, the information shared among authorized individuals is secured. In addition, a blockchain can ensure that information in a PSC management system is consistent and reliable. Another critical aspect of blockchain is that information can be easily accessed from any part of the world. However, it does not mean that everyone can easily access the information. The security of blockchain is robust enough so that only authorized stakeholders can have access to it. Blockchain technology can be used to prevent the spread of counterfeit medicines [34]. Traceability mechanisms work highly efficiently with blockchain technology, and can therefore be used in PSCs to precisely monitor the interconnectivity of the chains. Thus, if there is any flaw that allows the use of counterfeit medicines, it can be countered more easily.

Glover and Hermans [39] reviewed the potential capabilities of blockchain technology, showing that they were able to improve clinical trials by ensuring that sufficient amounts of APIs present in the raw materials were sourced from different reliable suppliers, and by ensuring that all participating PSC partners recorded critical information regarding their medications in the shared network.

Phase 2: Manufacturers

*RFID*

Most pharmaceutical manufacturers prefer RFID technology because one of its distinctive features is that it is able to provide automatic identification. Manufacturers can tag their products with sophisticated RFID chips that contain electronic information, including a unique serial number, that helps them trace their medications [29] electronically.

Deisingh [40] proposed using RFID coupled with an EPC, similar to how barcodes are used in supermarkets. These are applied by manufacturers, and store and carry basic drug information given by the manufacturers to the authorized retailers through the distribution chains.

*Pedigrees and mass serialization*

Bansal et al. [4] pointed to pedigrees and mass serializations as other types of traceability technologies that manufacturers use as part of their encoding processes to ensure the integrity of delivered drugs. Deisingh [40] explained how the global trade item number (GTIN) and the serialized global trade item number (sGTIN) systems, which are two types of mass serialization, can be used as ways to manage traceability, ensure safety, and systematically recall counterfeit medications.

*Blockchain*

Key pharmaceutical manufacturing companies, such as Genentech, AmerisourceBergen, McKesson, Pfizer, and AbbVie, agreed on the MediLedger project in 2017, which is based on implementing blockchain technology that would provide them with the ability to trace their products from the production stage to the quality control and distribution stages [41]. Schöner et al. [42] proposed a drug security prototype based on blockchain technology solutions that would enable them to establish transparent traceability and allow manufacturers to trace their medications. On the other hand, Huang et al. [43] proposed a drug traceability system for manufacturers based on blockchain to trace their products and identify their locations.

Tseng et al. [21] outlined a database called Gcoin, which is based on blockchain, and which can store medication information for all of the authorized parties to a PSC in order to control the sourcing of resources without the need for a centralized government entity. Kumar et al. [44] proposed a private system based on blockchain technology that would be managed by manufacturers, and which would provide real-time traceability of medications at any stage of the PSC. Kumar and Tripathi [45] proposed a drug traceability model based on blockchain technology that would be encrypted with a quick response (QR) security code to detect the correct APIs during the manufacturing stage to prevent counterfeit drugs from being introduced into the PSC. Bryatov and Borodinov [25] proposed a blockchain-based business model called Hyperledger Fabric that can assist manufacturers in tracing their products and eliminating any penetration of counterfeits in legitimate supply chains. Abbas et al. [26] proposed a novel blockchain system that can be deployed with Hyperledger Fabrics and which is based on machine learning technologies. This system can be used for drug traceability to detect the presence of counterfeit medications in a smart pharmaceutical industry. Shahbazi and Byun [46] looked differently at enhancing the security of system transactions and protecting the underlying data related to the manufacturing processes by proposing an integrated framework of blockchain and machine learning to improve the smart manufacturing of drugs.

Phase 3: Packagers

*RFID*

Kumar and Tripathi [45] discussed RFID and barcodes and listed their usages by packaging companies where they are attached to medication bottles specifically to verify the legitimacy of drugs.

*Barcodes*

Gautam et al. [47] discussed using simple barcode technologies by packagers to tag drug packages and prove product authenticity. Venhuis et al. [2] suggested using unique 2D barcodes on all medication packages to simplify the scanning and reveal the history and other critical information related to the medications.

Klein and Stolk [48] highlighted another type of barcode known as a 2D data matrix, which can capture electronically encoded information, such as manufacturer ID, product ID, or a unique ID used commonly on drug packages.

Phase 4: Distributors/Wholesalers

*RFID*

Sharifian et al. [49] stressed the importance of implementing RFID technology to scan and trace information about medications to ensure the authenticity of drugs during the transportation stage. Nilsson et al. [50] proposed a novel method for implementing active RFID technology that is embedded with time-controlled numeric tokens (TCNT) which, when combined with the seal of drug products, would ensure the security of medications through the logistic chain, especially at its weakest point when it is with the distributors.

*IoT*

Ng et al. [51] explored the essential uses of the internet of things (IoT) for pharmaceutical logistics. IoT systems can be coupled with RFID sensors, 2D bar codes, or smart labels to track drug shipments, trace high-value medications and validate authenticity. Singh et al. [3] proposed another use of IoT sensor-based blockchain, showing how it can be used to track and trace medicines during their transportation, as well as closely monitor their temperature and prevent the penetration of counterfeit drugs in the legal chains.

Phase 5: Hospitals and Pharmacies

*RFID*

Coustasse et al. [7] discussed the benefits of RFID technology for a hospital supply chain, where its implementation could help to improve service quality, reduce costs, monitor the temperature of perishable medications, and track hospital inventories in real time to ensure the punctual availability of medications for patients. Martínez et al. [52] showed the importance of implementing RFID technology to trace high-risk and high-cost medications in order to ensure the safety of drug prescriptions given to patients in day hospitals. Del Carmen León-Araujo et al. [53] showed how RFID technology could be embedded in smart cabinets to manage the circulation of drugs inside smart hospitals.

*Blockchain*

Thatcher and Acharya [54] proposed an electronic prescription model called the prescription drug monitoring system (PDMS). The system is based on blockchain technology, and aims to solve the opioid crisis by introducing a single point of failure. On the other hand, Jamil et al. [55] outlined another version of an automated drug prescription system that has been proposed by Hyperledger Fabric which would ensure the safe circulation of prescribed medications within the different sections of smart hospitals.

*IoT*

Iqbal et al. [56] outlined the architecture of a new approach to monitoring the health of elderly patients. The approach is based on an intelligent task-mapping system for a closed-loop IoT healthcare environment where the system could detect and provide notifications of any abnormalities, such as the provision of the wrong medication or deteriorating conditions, which would lead to faster interventions and assistance. The same authors proposed another use of IoT technology that can ensure the safety of patients with chronic diseases or other fatal diseases through the administration of a smart patient health monitoring system (PHMS), which is based on an optimized scheduling mechanism that uses IoT tasks [57].

Phase 6: Patients

*Smartphones*

Huang et al. [43] argue that mobile phones are small, user-friendly portable devices that can simplify the traceability process by helpings patients verify the authenticity of their medications. Table 2 shows a detailed summary of the implementation of traceability technologies in various phases of the PSC from the literature, provided in a tabulated form for easy reference.

### 3.3. Barriers to Establishing a Safe Pharmaceutical Supply Chain

Although the implementation of traceability technologies hugely contributes to detecting counterfeit drugs, there are barriers which prevent the complete establishment of a safe PSC. Table 3 shows a summary of these barriers based on the literature.

*PSC complexity*

The complexity of the PSC is caused by the fragmentation [8,59] of the pharmaceutical system, where the drug passes through different stages, channels, and partners. It is not easy to apply standardization and common codes [58] between individuals of the supply chain and oblige them to open communication channels, especially if they come from different backgrounds. In addition, this type of supply chain is temperature sensitive, making it very challenging to keep medications usable and effective within a specific temperature range [3].

*Insufficient regulation*

Improper rules and regulations, unstable policies, and a lack of common codes will eventually affect the safety of the PSC and patients [2,7,9,44,58,59,60].

*Lack of visibility and transparency*

Lack of visibility may happen in different areas, such as regulations, stock [8,58], collaborative relationships, mutual understandings, corruption of individuals [6], and unclear traceability [44,59].

*Cost issues*

Costs are associated with the production, supply, inventory, and procurement of drugs [8,44]. Other costs include the higher price of healthcare [6] and health insurance [61], different rates of interest while purchasing medications [58], different currency rates, and high costs of developing a modern traceability technology to detect counterfeit drugs [4,8,44]. There is also an additional cost applied to the original price of the medicines to cover the overhead costs, logistics costs, plus a profit, usually referred to as a mark-up [62].

*Lack of information and effective communication*

Delays in sharing information [8], sharing less or more information than needed [44,58,59], and the breakdown of the IT infrastructure cause the loss of information, especially if it is not secured properly [60].

*Poor quality or insufficient availability of raw material*

Adulterated or degraded raw materials [8,44,58,63] or even the poor quality of shortlisted suppliers [60] can adversely affect achieving a safe PSC.

*Lack of knowledge regarding the manufacturing process*

Insufficient production capabilities [60], unavailability or scarcity of skilled labor required for quality manufacturing [8,58,61], disruption in the production process, or machine failures [44] can all affect the safety of medications that drug manufacturers produce.

*Improper packaging*

Pharmaceutical packaging protects medications from exposure to factors that cause environmental deterioration, such as cold and heat, as well as from counterfeiting [31]. Errors when packing drugs with technology-protected packs [48], incorrectly printed details [58], or the reuse and relabeling of previously salvaged packages that have been recovered after selling authentic drugs will negatively affect the safety of the purchased medications [2,59,64].

*Improper storage*

The inadequate storage of drugs or improper maintenance of cold chains [2,58,60] will quickly degrade the current APIs and lead to adulterated medications. The lack of appropriate storage facilities [44] to store drugs according to standards can affect the safety of end-beneficiaries.

*Poor logistics and distributions*

Outsourcing logistics activities to unreliable transporters [65] can have dire consequences for the safety of the PSC [8]. Other factors can also affect drug distributions, such as crowding, accidents, and bad weather [58], or issues related to designing transport networks [60].

*Patients’ lack of knowledge*

The point of consumption is considered to be the most vulnerable stage for the entry of counterfeit medications, especially if patients lack the knowledge and the technology to assist them in detecting counterfeit drugs. If it is difficult for even knowledgeable doctors and pharmacists to detect fake medications, patients will certainly find it much harder [59].

*Rise in online purchases*

The extended use of the internet has created an unsafe supply of medications via simplified online purchases [59]. Typically, patients opt for cheap alternative sources where they can purchase medications with attractive offers and volume discounts from fraudulent online suppliers [68]. Many patients target these sources to avoid the need for prescriptions [67] or to purchase expensive medicines at lower prices [66]. The quality of medications that are purchased online is questionable and threatens patients’ health and safety [69].

*External influences*

Some factors that are outside the control of people involved in the PSC can also disturb the safety of the PSC, such as natural disasters [8], climate disturbances [44], terrorism, or political issues, such as wars or sanctions imposed on certain countries [60].

### 3.4. Critical Success Factors for Achieving a Safe Pharmaceutical Supply Chain

Implementing traceability technologies in the PSC can provide more than just a perfect means of traceability. They will be able to detect the falsified medications that penetrate the legitimate supply chains if these technologies are implemented with an array of success factors in mind. Table 4 summarizes the shortlisted critical success factors for achieving a safe PSC.

*Collaboration*

According to scholars and professionals, collaboration remains a vague concept that is challenging to implement unless performed correctly [71]. Min et al. [72] defined collaboration as the sharing of information, benefits, and risks, which is the “driving force” behind achieving effective supply chain management and the basis for internal and external communication and the reinforcement of traceability. Collaboration should be of the right amount and tied to definite activities in the supply chain in order to achieve the maximized benefit because the number of individuals involved in each activity differs according to each phase of the supply chain. Most researchers consider collaboration to be an integral part of the culture, involving trust, information sharing, cooperation, and coordination.

a. Culture

A working environment that supports collaboration is essential. Without this cultural understanding, collaboration will be difficult to implement in a supply chain [71].

b. Trust

Trust between PSC partners is critical to achieving successful long-term relationships [16].

c. Sharing information

Sharing information is an essential ingredient for day-to-day collaborative operations [72]. The willingness of supply chain parties to exchange ideas, data, and collaborate is essential to improve partnerships in the supply chain [71].

d. Cooperation

Cooperation requires maintaining good relationships within an organization and with other partners in the supply chain [28]. It depends mainly on proper communication among supply chain partners, which helps improve inter- and intra-organizational relationships [81].

e. Coordonation

Coordination includes sharing resources and closer cooperation among the supply chain actors to improve value chain procedures [81].

*Visibility and Transparency*

This is “the ability to see transparently through all supply chain links” [16]. Visibility and transparency enable partners within the supply chain to disclose information [80], share a mutual understanding, and reduce information asymmetry so that all can access the same information simultaneously. Papert et al. [77] classified the pharmaceutical supply chain’s visibility into four dimensions: availability, identity, position, and status quo.

*Government support*

Governments monitor the manufacturers, inventories, and distributors, and determine the legal use of these medications, as well as the proper disposal processes of recalled medications [28]. Fadlallah et al. [12] pointed to the importance of governments when raising public awareness in order to eliminate purchasing medications from illicit sources, such as online pharmacies, to maintain public safety.

*Quality of information*

Partners of the PSC always expect to receive high-quality information or products [16]. If the shared information is incorrect at any point, this could adversely affect the safety and security of patients.

*Integration*

Integration “represents mutual understanding, a common vision, shared resources, shared risks and achievement of collective goals” intra-organizationally and inter-organizationally [70]. De Vries and Huijsman [75] stated that the integration of health supply chains occurs in areas such as processes in general, planning processes, intra- and inter-organizational information flow, market approaches, and market development. More focus should be given to integrating processes in order to gain better performance of the PSC and ensure the safety of drugs.

*Stakeholder support*

Establishing a healthy relationship between stakeholders is central for maintaining trust and support for collaboration in a PSC [73].

*Cost Effectiveness*

Cost effectiveness is expressed in different forms, especially when implementing a traceability technology. The focus is typically on price reduction, process improvement, or on increasing the quality of extracted data, consequently increasing the ability to detect any disturbances to the legitimate supply chain and ensuring safety [17].

*Resource management*

Investing heavily in human resources is a critical factor for achieving safety in the supply chain, such as forming the right teams [74] and providing adequate training to individuals [76] to check the authenticity of the received raw materials from suppliers and follow good manufacturing practices (GMP) as per international standards [8].

*Data security*

Ensuring data security and software privacy is essential to guarantee the credibility of the platform and the privacy of users in order to earn their trust [79].

*Dedicated IT infrastructure*

The base platform ensures the safety and security of the medication information that is exchanged between PSC partners when implementing proper traceability technology. IT infrastructure helps to sustain, deploy, and leverage different resources to standardize communication between all parties [81].

*Decentralized system*

The traditional supply chain was based on a centralization approach, where the authority was centered around a specific individual or section of the supply chain. One main reason for implementing traceability technology in PSC is to gain decentralization as a critical success factor in order to avoid dependence on a single controlling authority [82]. Traceability technology will also increase collaboration without trust between all supply chain partners [78].

## 4. Discussion

This section summarizes, discusses, and analyzes all the data gathered from the literature review. As discussed in the earlier sections, counterfeit medications are significant issues and challenges for a legitimate PSC. The safety and security of drug products are gaining global attention, especially with the increased counterfeit drug incident cases worldwide.

### 4.1. Conceptual Framework and Guidelines for a Safe PSC

Counterfeit drugs typically enter the market through the gaps in the PSC and can be inserted during any phase where they are either mixed with the original drugs or brought in on their own. According to the research, most companies depend on API-producing countries, such as China and India, that export them based on market needs. Due to the high market demand, suppliers of raw materials often search for separate companies that offer the raw materials and who will agree to place their labels on the products, but this poses a significant problem for product quality. The raw materials suppliers may then have a limited time to check a product’s quality before it reaches the market.

Raw materials are manufactured and transported from one country to another through various distribution methods. Many additional processes might occur during the distribution process, such as repackaging the products for better shipment. This is an ideal chance for a counterfeiter to easily alter the original products.

**Figure 9:**
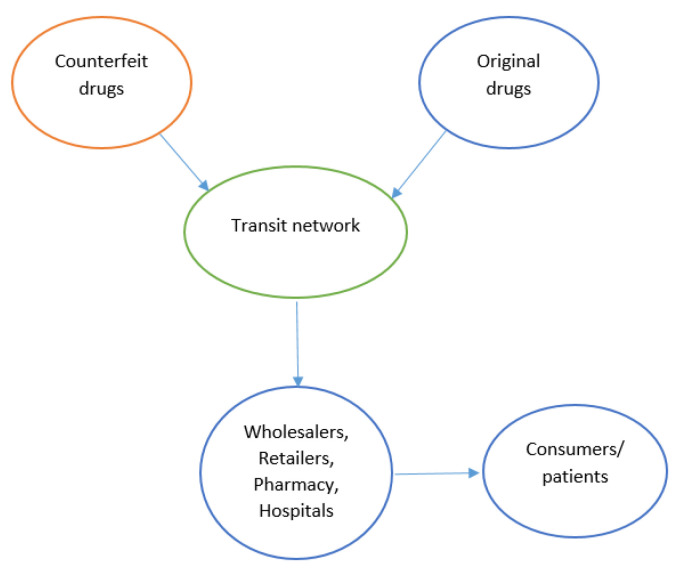
Entry of counterfeit drugs in the PSC.

Wholesalers indirectly contribute to the entry of counterfeit products into the market. Counterfeit products find their way into the market through arbitrage. Arbitrage is the act of buying a product in one market and then reselling it at a higher price in another market, hence making a profit from the cost difference. Wholesalers often buy the drugs and then sell them in the market where the prices are high. These drugs could be cheap and counterfeited and purchased from different countries. Figure 9 illustrates the two different methods for the entry of counterfeit drugs in the legitimate PSC.

Because counterfeit medicine threatens people’s health, the WHO helps ensure access to safe and quality medicines everywhere by supporting regulatory changes and improving technical capabilities. However, with increased technology and globalization, the problem will grow continuously unless several solutions are applied. Among these solutions are the following:

1. A PSC should be legitimate, regulated, and licensed by the health ministry or health body to supply authentic drugs to patients.
2. In case of any suspicious activity concerning the packaging, pill size, color, texture, or taste compared to the previous prescription, the patient should report the matter or contact the manufacturers or the physician because these might be counterfeit medications.
3. Healthcare and pharmacists can also assist patients in preventing counterfeit drugs from entering the PSC by purchasing from reliable and known sources. They can also warn patients against buying their medication online by telling them the dangers that they may encounter.
4. Being cautious when shopping for medications online. Always shop in approved online pharmacies that display a Verified Internet Pharmacy Practice Sites (VIPPS) seal. Sites without this seal often sell counterfeit drugs.
5. Implementing an effective PSC security system with several protective measures.
6. Recommending ways in which health authorities can detect and deal with counterfeit drugs in the industry.
7. Reaching a global common understanding to create awareness of what counterfeit drugs are.
8. Developing a national action plan that creates prevention and detection measures to respond to counterfeit drugs.

**Figure 10:**
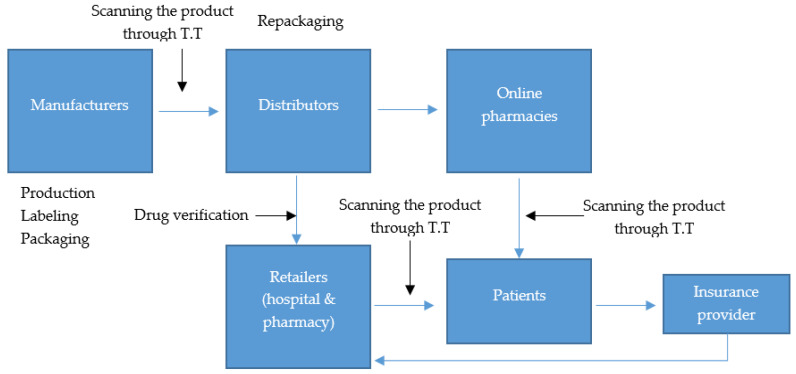
Framework for a safe PSC.

Figure 10 illustrates a framework for achieving a safe PSC by demonstrating the path drugs need to follow to ensure their safety.

Manufacturers:

Entities receive raw materials from authorized suppliers of raw materials and produce finished drugs in the correct quantity and quality. Finished drugs are then packaged, labeled with the manufacturing company logo, and prepared for distribution.

According to the review, packaging and labeling play a critical role as a first step in thwarting counterfeit drugs. Labeling also helps to protect the trademark and the intellectual property rights of the manufacturing companies. Some anti-counterfeit measures that can be used with labeling and packaging include:

*Watermarks*

These are images or patterns embedded into the design that the manufacturers use to authenticate the product and protect their brand [40]. It is usually very hard to notice or duplicate a watermark.

*Micro text*

Micro texts are tiny codes or symbols that are hard to replicate without advanced detection and printing equipment [16]. Counterfeiters have difficulty seeing these micro texts with the naked eye and are usually unaware that they exist.

*Holograms*

This is a three-dimensional image formed by the interference of a beam of light. Holograms incorporate the various forms of data and then produce tracking information.

*RFID system*

This technology uses radio waves and helps identify a product, such as drugs, by transmitting the information on a microchip attached to the antenna [29]. It detects valuable information concerning the product and can help identify and detect counterfeit products. The finished drugs can be scanned using RFID technology before being received by distributors to ensure authenticity.

Distributors:

Distributors acquire or receive the finished drugs from the registered manufacturers. They usually act as intermediaries between the manufacturers and their customers. Distributors can decide to repackage the drugs that they acquired from the manufacturers. This then presents another opportunity for counterfeiting to easily occur. To prevent this, after repackaging, all the drugs should be scanned using traceability technology, such as RFID, and verified to detect the falsified ones before they reach pharmacies or hospitals.

Online Pharmacies:

Online pharmacies are companies that sell drugs legally or illegally through the internet. The extended use of the web has simplified the operation of online trade, especially for expensive medications that patients are unable to purchase through legal sources. Online pharmacies also receive the drugs from various distributors and sell them directly to the patients. According to the review, online pharmacies have proliferated over the years. This growth has led to another opportunity for counterfeit drugs to find their way into the market. Most patients purchase drugs online blindly without considering whether the sellers are certified or not. The patients are also at risk because they cannot tell whether the vendors are offering the same quality of drugs as those offered in a retail pharmacy. To prevent more risks, legal online pharmacies should be licensed in the area that they operate, and they should have a VIPPS logo on their site, which shows that they are licensed [67]. Medications purchased online should also be scanned through traceability technology to ensure that they are not counterfeit. Patients should be knowledgeable of what to look at when shopping for their drugs online.

Retailers:

Retailers are pre-authorized entities that provide or sell prescribed medications to patients. According to the review, most patients receive their prescribed treatments from hospitals. Patients trust all kinds of drugs that they receive from the hospitals and believe that hospitals do not provide counterfeit products. Therefore, hospitals should apply traceability technology and scan their products to ensure product safety before providing them to patients [59]. They should also ensure that their suppliers are authorized and trusted and never supply falsified drugs.

Patients:

Patients are the ultimate beneficiaries of medication products and are entities who purchase, consume, or store medications. Counterfeit products usually significantly affect end users. According to the review, they usually get into their hands without their knowledge or because of ignorance, or because they trust their drugs providers. To protect the patients from these falsified products, they should be trained on how to identify them and on what to do if they find or become suspicious of a particular drug being falsified or note a difference from the previous medication. They should always report the matter to their physician and be careful when buying their medication online.

Insurance provider:

The role of the insurance provider is to reduce risks that might affect patients and prevent the spread of counterfeit drugs. They usually have a potent influence as a buyer of health services. Insurance providers can also reduce the financial loss in case of any catastrophic occurrence during illness [29]. They should then ensure that the patients are well covered even in the event of utilizing counterfeit products.

**Figure 11:**
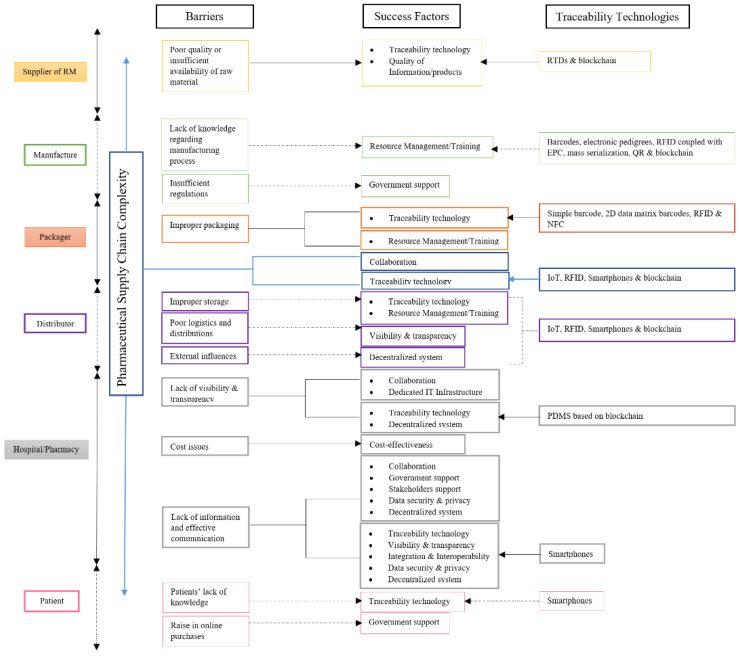
Guidelines for a safe PSC.

Figure 11 illustrates guidelines for achieving a safe PSC by demonstrating the influence of traceability technologies and success factors on overcoming the various barriers in different phases of the PSC.

Phase 1: Suppliers of raw materials

Poor quality raw materials are a barrier at the supplier’s phase. Implementing traceability technology and ensuring the quality of information or products are the success factors used to overcome this barrier. Along with the various traceability technologies reviewed, blockchain is the most suitable one to overcome this barrier. A blockchain can be used as a single point to store important information about purchased raw materials from different suppliers and ensure that they contain the correct APIs.

Phase 2: Manufacturers

Two barriers affect the drug manufacturing phase. The first one is the lack of knowledge regarding the manufacturing process. The most significant enabler would be properly managing the resources and ensuring the intense training of the workforce when installing and using traceability technologies of all kinds, such as barcodes, mass serialization, and RFID, to detect counterfeit drugs.

The lack of enough regulation is a second barrier that leads to unregulated manufacturing processes. Government funding and support can encourage the implementation of traceability technology to detect counterfeit drugs.

Phase 3: Packagers

The improper packaging of medications affects safety at the packaging phase. There are two factors for successfully overcoming this barrier. The primary one is implementing traceability technologies to ensure visibility and real-time status updates, which assist in rapidly detecting any irregular act. Among all traceability technologies, RFID and sophisticated barcodes are considered to be the most effective ones. The other success factor is training human resources to perform the packaging process correctly, to deal with traceability technologies, and to install them precisely.

Phase 4: Distributors/Wholesalers

Inadequate storage adversely affects essential pharmaceutical compounds and degrades the APIs, which is a barrier in the distribution phase. There are two success factors to be considered for overcoming this barrier, i.e., traceability technologies and resource management training. RFID is the most suitable for providing clear visibility, temperature control, and real-time monitoring among the various traceability technologies. Then, the human resources in charge of the storage and transportation processes have to be well trained to retain the drug cold chain as recommended by the manufacturers and adequately use the implemented traceability technologies to detect counterfeits.

Poor logistics is another barrier that also affects safety and disturbs the continuity of drug distribution. The most important way of overcoming this barrier would be to have the required level of visibility and transparency during the logistics process. An emerging traceability technology can achieve visibility and transparency of the PSC, for example, blockchain. Blockchain assists in changing the traditional way of drug distribution to a more robust and transparent one by creating multiple points of records distributed in various locations visible only by authorized members in order to manage drug shipments in real time [26].

There are also uncontrollable external factors, such as natural disasters [8], environmental disturbances [44], and all kinds of political issues [60]. A decentralized system that is built on blockchain, which supports data immutability, can tackle these uncertainties. It can also avoid the single point of authority, responsibility, infrastructure, and failure, where only authorized members can access, monitor, store, or update information from their systems [21].

Phase 5: Hospitals and Pharmacies

Lack of visibility and transparency is one barrier that can significantly affect the safety of drugs as they reach hospitals and pharmacies. Certain critical success factors that need to be considered to overcome this barrier are as follows.

Collaboration between partners of the PSC can provide a mutual understanding and help to share information freely. Traceability technology can provide visibility, precise monitoring in real time, and effective communication between partners of the PSC. The most effective one would be utilizing blockchain to provide visibility to authorized members [79]. A dedicated and reliable IT platform through which traceability technology can be applied for transparent communication without the need for trust between the retailer’s supply chain partners is necessary. A decentralized system can be utilized for implementing a blockchain, which can provide the much needed visibility. Blockchain is also immutable unless changes are agreed upon by all partners [43]. Costs associated with healthcare are a source of fear for patients and another barrier that affects the same phase. Cost efficiency is the critical success factor in its various forms, though it is not necessarily tied to reducing costs. However, it can include improving the current process, increasing the quality of shared data, and adapting the right traceability technology to detect any disturbances in the legitimate PSC.

The lack of exchange in information and effective communication is also a barrier that affects hospitals and pharmacies. There is a group of critical success factors to be considered that can help to overcome this barrier, and they are as follows.

Collaboration between partners within the supply chain and their willingness to communicate effectively can act as an essential success factor [83]. The proper selection of traceability technology will ensure effective communication between the supply chain partners where all shared information is being monitored, tracked, and secured [16]. Visibility and transparency of the information are required to a certain extent where they provide mutual agreements and reduce the asymmetry of the shared information. Government support and funding are also essential to overcome this barrier. System integration and interoperability are critical for process unification and mutual understanding between partners of the PSC, because this would ensure the effectiveness of the information sharing [84]. The support of stakeholders and their willingness to collaborate is a critical factor in achieving successful communication. Data security and privacy are also mandatory to overcome the lack of effective communication and reinforce trust. A dedicated IT infrastructure can serve as a reliable platform for effective and secure communication between the supply chain partners. Establishing a decentralized system through a blockchain platform facilitates communication efficacy and trust between supply chain partners, helping authorized members share required information from different locations [85].

Among the various traceability technologies, blockchain can prove its effectiveness by providing much needed collaboration, visibility, and transparency, as well as data security to overcome the communication discrepancies in the PSC.

Phase 6: Patients

The patient phase is considered to be the most vulnerable stage for any counterfeit medication entry, especially if patients lack the knowledge and the proper technology to detect a counterfeit. The most influential success factor for overcoming this barrier includes traceability technology in patients’ smartphones that can help verify the legitimacy of their medications on their own.

The growth in online pharmacies adversely affects patients’ decisions and permits them to search for cheap sources from which to purchase their medications. Governments must support patients by setting strict regulations to control online pharmacies, publishing a government-approved list, and controlling medication prices to stop the manipulation of generic and branded drugs and ensure medications’ cost effectiveness.

All Phases (PSC complexity)

One significant barrier affecting the PSC is the complexity and fragmentation [8] of the pharmaceutical system, where the drug passes through different stages, with its ownership changing all the time. This barrier affects all phases of the PSC and is not limited to one phase. There are two success factors to be considered to overcome this barrier. The first is to understand the complexity of collaboration between partners in the PSC and manage it properly [86]. The second is implementing traceability technology or upgrading a current one to overcome the complexity barrier and limit the spread of counterfeit medicines. Among the various traceability technologies, blockchain is the most influential one to overcome complexity, improve visibility [87], facilitate collaboration among business partners, and improve the overall performance of the supply chain.

A safe PSC will reinvigorate pharmaceutical organizations, helping them better use their assets and resources and boosting the values and profits for shareholders, while still fulfilling the patients’ demands.

To ensure the safety and availability of useful drugs for patients, the PSC should focus on several steps, which are:

- Connecting and collaborating with the patients and the supply chain partners in order to provide end-to-end visibility and different business interactions;
- Closely monitoring the manufacturing process to ensure product consistency, making sure that drugs are not contaminated, because an extra substance in the manufacturing process may be life threatening to the end beneficiaries;
- Having end-to-end traceability;
- Having a quick response to a change in demand. Responding to demand changes quickly and coming up with the best solutions is important in the pharmaceutical industry; and
- Having complete inventory visibility. Drug manufacturers should ensure complete downstream inventory visibility with good shipping practices. This eventually ensures that the products are reaching their intended destination.

### 4.2. Overall Findings

The first key research question that the study has answered is related to applying traceability technologies to various PSC phases to detect and solve counterfeit issues. In order to answer this question, a comprehensive review was conducted that found key studies which explained in detail the application of traceability technologies in various PSC phases to detect counterfeit drugs. With the help of the literature, six phases were identified: the raw material supply phase, the manufacturing phase, the packaging phase, the distribution/wholesale phase, the hospital and pharmacy phase, and finally, the patient phase. Each phase was found to be essential for ensuring that counterfeit issues are detected and solved. The pharmaceutical market experiences a lot of adulteration and forgery due to the high revenue gained from illegal trafficking. Hence, the importance of tracing drugs was discussed in the study throughout all the stages of the PSC, from the raw materials to the consumption by patients. The traceability technologies identified included all types of barcodes, RFID, blockchain, IoT, pedigree, and mass serialization. The latest statistics in the pharmaceutical sector showed that fake medications would continue to penetrate legitimate PSCs at any weak points due to the desperate need by different patient segments. Theyel [30] implied that the tracing of drugs throughout all the stages of the PSC should be conducted to detect counterfeit drugs and ensure a safe PSC.

The second research question had two parts that this study answered in detail. First, what are the various barriers affecting the establishment of a safe PSC? To answer this question, the study conducted an extensive review of the literature and found that the complexity of the PSC was the most significant barrier affecting the PSC due to its effect on all phases of the supply chain. Complexity is the main cause of fragmentation in the pharmaceutical system. Having individuals and stakeholders from different backgrounds increases the complexity of establishing trust and open communication channels. Other barriers identified included the failure to have enough regulations, lack of information and effective communication, lack of visibility and transparency, high-cost issues associated with healthcare, impaired quality of raw materials, manufacturing, packaging and storage, and poor logistics that will all lead towards failure in the attempts to establish a safe PSC. Hence, the study has answered the question by identifying barriers which affect the establishment of a safe PSC.

The other part of the question was to assess the critical success factors that will help overcome the barriers to establishing a safe PSC. The study also conducted an extensive review of the literature to answer this. The critical success factors identified were collaboration or its drivers between the partners, visibility and transparency, government support, quality of information or products, integration and interoperability, stakeholder support, cost effectiveness, resource training, data security, dedicated IT infrastructure, and decentralized systems. Likewise, based on the findings related to the traceability technologies that could have a significant influence on overcoming the barriers to establishing a safe PSC, implementing these critical success factors has equal importance. However, implementing traceability technologies in the PSC is insufficient to provide the perfect means of traceability and detect the falsified medications that penetrate the legitimate supply chains unless these technologies are led by success factors that support the means for their implementation.

### 4.3. Managerial Implications

Based on the findings of this study, certain decisions can be taken related to the safety of the PSC. Managers in pharmaceutical sectors should ensure that they select the proper combination of traceability technology and the appropriate critical success factors to detect counterfeit drugs and simultaneously overcome barriers.

All technologies have advantages and disadvantages, and some perform better than others in certain situations depending on the product type and the requirements [88]. Findings revealed that simple or sophisticated barcodes, mass serialization, and RFID systems could all prove effective in detecting counterfeit drugs in the PSC. Ng et al. [51] gave an opposing perspective; they argued that simple barcodes and RFID on their own prove ineffective, which is why emergent blockchain-based traceability solutions are becoming more influential. They bring transparency to the supply chain [89], preserve the history from their point of origin, ensure immutability [90], and have more applicability in almost all stages of the PSC and even for monitoring vital signs. Beginning with the raw material and sourcing stage, the findings show that blockchain technology is the most effective solution for ensuring the purchased raw materials contain the correct APIs [32]. Counterfeiting issues are most pronounced in the manufacturing stage, and blockchain solutions in the form of Hyperledger Fabrics have proven to be the most effective solution to drug counterfeiting [26,41].

Among the key barriers identified that can lead to issues of drug counterfeiting are complexities, the high cost of healthcare, lack of knowledge, and online purchasing. Most studies have identified these issues as the most persistent. Considering these barriers, leaders of the PSC have to act in the interest of patients, invest in training their workforce to handle medications at different stages properly, and establish technologies that help stakeholders collaborate and share information, and which give them much needed visibility. Pharmaceutical trade regulations should be revisited, and strict rules applied to protect all segments of patients from increases in medication and healthcare prices. Healthcare and pharmacists can also help patients to avert counterfeit drugs by buying from trustworthy and known sources. They can also advise patients not to purchase their medications online by discussing the hazards they might encounter unless these websites have the VIPPS logo.

The study has also identified certain critical success factors that support achieving a safe PSC, and findings have revealed that collaboration, visibility, transparency, and government support are the most influential factors for overcoming barriers and ensuring an effective PSC. However, other studies provide different perspectives in this regard. Karlsen et al. [76] argues that collaboration and government support are the most effective success factors for detecting counterfeits and ensuring a safe PSC, but Tian [78] believes that only visibility and transparency are the way for forward, and attributes little significance to data security. De Lima et al. [16] highlighted the need for collaboration at a trust-based level between stakeholders of the PSC to ensure a higher quality of medications and ideal methods of eliminating counterfeit drugs. Although this solution is applicable, companies working via online platforms require much more than just trust between PSC stakeholders to collaborate. Although critical success factors are essential to identifying and eliminating counterfeit drugs, and ensuring a safe PSC, situational conditions and subjective approaches are more important to identify the most applicable strategies.

### 4.4. Theoretical Implications

In a world where the environmental, economic, and social domains are increasingly complex, digital technologies have become a go-to option to ameliorate the looming threat of socioeconomic and environmental crises [31]. Because supply chains are the jugular vein of a modern globalized economy, their efficacious functioning is paramount to success, especially with an imminent environmental crisis. As shown in this study, threats to data security, lack of transparency, inconsistencies in product pricing strategy, and lack of visibility of materials in between the supply chains are major factors behind the burgeoning problem of counterfeit products [91].

Most previous published papers that were reviewed focused on identifying the type of technologies useful for traceability and detecting counterfeit drugs for a particular phase of the PSC. Other papers showed only critical success factors or factors that hindered the establishment of a safe PSC. The current study differs from others and contributes to the current literature based on the three objectives discussed, as it integrated and showed the important links among the traceability technologies, critical success factors, barriers, and various phases of the PSC to detect and solve counterfeit issues, especially because the influence of all three together was not considered previously, and their influences on each other and the different phases of the PSC were not analyzed. This was illustrated in the form of guidelines for achieving a safe PSC.

### 4.5. Limitations and Scope of Future Research

Time constraint was the key limitation associated with this study. The time allotted was insufficient for covering a broader array of literature. Thus, more studies could be reviewed to obtain more information. In addition to this, the study examined only secondary data published in previous papers.

Although this review encompassed a large domain of traceability technologies and their influence on ameliorating barriers in PSCs, some other barriers could not be solved through such techniques. With the imminent climate crisis, the sustainability of supply chains remains a question mark for manufacturers, retailers, distributors, and policymakers. The literature is scarce regarding the impact of novel traceability technologies introduced to improve the transparency of drug transportation networks. In the presence of a highly fossil fuel-based supply chain, the current review does not address the environmental and social impacts of these new technologies. Furthermore, the current drug regulatory framework in the majority of the countries is arduous, which concomitantly has left loopholes in the process. Counterfeiters exploit these loopholes to leak their counterfeit products into the formal market. Because counterfeiters could also acquire traceability technologies through the same loopholes, studies on improving regulations are needed, which are not part of this review paper.

## 5. Conclusions

The health crisis that the world went through recently has adversely impacted the pharmaceutical sector and seen an increase in counterfeit drug. Yet it further opened new opportunities to utilize various technologies to combat counterfeited products. Although healthcare techniques and the use of technology within the pharmaceutical sector have advanced, certain issues persist. Especially in the post-COVID-19 era, healthcare professionals face increasing burdens and burnouts. The high demand for drugs has created incentives for counterfeit drug manufacturing and inclusion into legitimate supply chains. Hence the purpose of this review was to highlight the importance of achieving a safe PSC and eliminating fake drugs from legitimate supply chains.

Recent statistics show that counterfeit drugs will continue to penetrate the markets which have weak or uncontrolled PSCs and target more significant patient segments. This review on the various applications of traceability technologies in different phases of the PSC can assist in detecting drug counterfeits. The most important is blockchain technology in the raw materials supply phase, RFID and mass serialization for manufacturers, RFID and barcodes for packagers, RFID for distributors, RFID and blockchain for hospitals and pharmacies, and smartphones for patients.

Although the implementation of traceability technologies contributes to detecting counterfeit drugs, some barriers restrict the entire establishment of a safe PSC, such as the complexity and the fragmentation of the PSC, insufficient regulations, lack of visibility and transparency, high healthcare costs, the impaired quality of raw materials, manufacturing, packaging and storage, and poor logistics. On the other hand, some factors support establishing a safe PSC, such as collaboration between the partners, visibility, government support, resource training, data security, and a dedicated IT infrastructure.

Finally, the growth of counterfeited drugs leads to the paramount need for further research in this area, mainly empirically testing the effect of traceability technologies and critical success factors for overcoming the various barriers to establishing a safe PSC. This study developed a conceptual framework and guidelines for achieving a safe PSC by demonstrating the influence of traceability technologies and success factors on overcoming the various barriers in different phases of the PSC.

Future researchers can focus more on the quantitative side of the study and conduct a statistical analysis to test hypotheses drawn from the current study. This can be achieved by surveys and interviews from respondents from the pharmaceutical sector to offer more comprehensive results related to the study’s topic. Other studies might include exploring the reverse PSC or testing the same framework for other products critical to human life, such as food, to create safe supply chains in other areas.

## Figures and Tables

**Table 1:** Identification of keywords.

Keywords	Search Strings	Databases
1. Traceability technologies to detect counterfeit drugs in the pharmaceutical supply chain	Traceability technologies AND Drug counterfeiting detection AND Pharmaceutical supply chain	Scopus EBSCOhost Science direct Emerald insight Taylor & Francis Online Springer search IEEE
2. Barriers to detecting counterfeit drugs in the pharmaceutical supply chain	Barriers (OR risks) AND Drug counterfeiting detection AND Pharmaceutical supply chain	
3. Critical success factors for detecting counterfeit drugs in the pharmaceutical supply chain	Critical success factors (OR success factors) AND Drug counterfeiting detection AND Pharmaceutical supply chain	

**Table 2:** Traceability technologies in various phases of the PSC.

Authors		Suppliers of Raw Materials	Manufacturers	Packagers	Distributors/Wholesalers	Hospitals/Pharmacies	Patients
	Phases						
Deisingh [40]			RFID coupled with EPC Pedigrees and mass serialization				
King and Zhang [29]			RFID				
Gautam et al. [47]				Simple barcode			
Nilsson et al. [50]					RFID		
Coustasse et al. [7]						RFID	
Sharifian et al. [49]					RFID		
Martínez et al. [52]						RFID	
Glover and Hermans [39]		Blockchain					
Schöner et al. [42]			Blockchain				
Huang et al. [43]			Blockchain				Smartphones
Klein and Stolk [48]				2D data matrix			
Ng et al. [51]					IoT sensors coupled with RFID		
Thatcher and Acharya [54]						Blockchain	
Tseng et al. [21]			Blockchain				
Venhuis et al. [2]				Unique 2D barcodes			
Bryatov and Borodinov [25]			Blockchain				
Del Carmen León-Araujo et al. [53]						RFID	
Jamil et al. [55]						Blockchain	
Kumar et al. [44]			Blockchain				
Kumar and Tripathi [45]			Blockchain with QR	RFID			
Woods and Iyengar-Emens [41]			Blockchain				
Abbas et al. [26]			Blockchain				
Singh et al. [3]					IoT sensors based Blockchain		

**Table 3:** Barriers to safe PSC.

**Barriers**	**References**
PSC complexity	Breen [58]; Jaberidoost et al. [8]; Tremblay [59]; Singh et al. [3]
Insufficient regulation	Breen [58]; Yu et al. [6]; Jaberidoost et al. [8]; Punniyamoorthy et al. [60]; Tremblay [59]; Venhuis et al. [2]; Kumar et al. [44]
Lack of visibility and transparency	Breen [58]; Yu et al. [6]; Jaberidoost et al. [8]; Tremblay [59]; Kumar et al. [44]
Cost issues	Breen [58]; Yu et al. [6]; Narayana et al. [61]; Bansal et al. [4]; Coustasse et al. [7]; Jaberidoost et al. [8]; Kumar et al. [44]; Lee et al. [62]
Lack of information and effective communication	Breen [58]; Jaberidoost et al. [8]; Punniyamoorthy et al. [60]; Tremblay [59]; Kumar et al. [44]
Poor quality or insufficient availability of raw material	Breen [58]; Jaberidoost et al. [8]; Punniyamoorthy et al. [60]; Li et al. [63]; Kumar et al. [44]
Lack of knowledge regarding the manufacturing process	Breen [58]; Narayana et al. [61]; Jaberidoost et al. [8]; Punniyamoorthy et al. [60]; Kumar et al. [44]
Improper packaging	Breen [58]; Tremblay [59]; Klein and Stolk [48]; Venhuis et al. [2]; Soon and Manning [64]; Salim et al. [33]
Improper storage	Breen [58]; Punniyamoorthy et al. [60]; Venhuis et al. [2]; Kumar et al. [44]
Poor logistics and distributions	Breen [58]; Jaberidoost et al. [8]; Punniyamoorthy et al. [60]; Singh et al. [65]
Patients’ lack of knowledge	Tremblay [59]
Rise in online purchases	Mäkinen et al. [66]; Gray [67]; Leontiadis [68]; Tremblay [59]; Shaikh et al. [69]
External influences	Jaberidoost et al. [8]; Punniyamoorthy et al. [60]; Kumar et al. [44]

**Table 4:** Critical success factors.

Success Factors for Ideal Traceability	Stank et al. [70]	Barratt [71]	Min et al. [72]	Doukidis et al. [73]	Fawcett et al. [74]	De Vries and Huijsman [75]	Karlsen et al. [76]	Fadlallah et al. [12]	Papert et al. [77]	Tian [78]	Khan et al. [28]	De Lima et al. [16]	Silva and Mattos [17]	Fernando [79]	Sodhi and Tang [80]	Hastig and Sodhi [81]	Rogerson and Parry [82]
Collaboration and its drivers Culture Trust Sharing information Cooperation Coordination	√	√	√	√	√		√	√			√	√	√	√		√	√
Visibility and transparency					√			√	√	√		√	√	√	√	√	√
Government support							√	√			√	√	√			√	
Quality of information/ products					√				√			√	√	√			
Integration and interoperability	√					√	√						√	√			
Stakeholder support					√		√	√			√		√				
Cost effectiveness					√							√	√	√			
Resource management/ training					√		√				√	√					
Data security and privacy								√		√		√		√			
Dedicated IT infrastructure					√						√						
Decentralized system										√							

## References

[1] Ghadge A., Duck A., Er M., Caldwell N. (2021). Deceptive counterfeit risk in global supply chains. Supply Chain Forum Int. J..

[2] Venhuis B.J., Oostlander A.E., Di Giorgio D., Mosimann R., du Plessis I. (2018). Oncology drugs in the crosshairs of pharmaceutical crime. Lancet Oncol..

[3] Singh R., Dwivedi A.D., Srivastava G. (2020). Internet of Things Based Blockchain for Temperature Monitoring and Counterfeit Pharmaceutical Prevention. Sensors.

[4] Bansal D., Malla S., Gudala K., Tiwari P. (2013). Anti-Counterfeit Technologies: A Pharmaceutical Industry Perspective. Sci. Pharm..

[5] Zhang H., Hua D., Huang C., Samal S.K., Xiong R., Sauvage F., Braeckmans K., Remaut K., De Smedt S. (2020). Materials and Technologies to Combat Counterfeiting of Pharmaceuticals: Current and Future Problem Tackling. Adv. Mater..

[6] Yu X., Li C., Shi Y., Yu M. (2010). Pharmaceutical supply chain in China: Current issues and implications for health system reform. Health Policy.

[7] Coustasse A., Tomblin S., Slack C. (2013). Impact of radio-frequency identification (RFID) technologies on the hospital supply chain: A literature review. Perspect. Health Inf. Manag..

[8] Jaberidoost M., Nikfar S., Abdollahiasl A., Dinarvand R. (2013). Pharmaceutical supply chain risks: A systematic review. DARU J. Pharm. Sci..

[9] Kovacs S., Hawes S.E., Maley S.N., Mosites E., Wong L., Stergachis A. (2014). Technologies for Detecting Falsified and Substandard Drugs in Low and Middle-Income Countries. PLoS ONE.

[10] Ab Talib M.S., Hamid A.B.A., Thoo A.C. (2015). Critical success factors of supply chain management: A literature survey and Pareto analysis. EuroMed J. Bus..

[11] Hamilton W.L., Doyle C., Halliwell-Ewen M., Lambert G. (2016). Public health interventions to protect against falsified medicines: A systematic review of international, national and local policies. Health Policy Plan..

[12] Fadlallah R., El-Jardali F., Annan F., Azzam H., Akl E.A. (2016). Strategies and Systems-Level Interventions to Combat or Prevent Drug Counterfeiting: A Systematic Review of Evidence Beyond Effectiveness. Pharm. Med..

[13] Sheikhi M., Goordarzi M., Shabani M. Key Success Factors of drug Supply Chain Performance. Proceedings of the International Conference on Industrial Engineering and Operations Management.

[14] Mackey T.K., Nayyar G. (2017). A review of existing and emerging digital technologies to combat the global trade in fake medicines. Expert Opin. Drug Saf..

[15] Ding B. (2018). Pharma Industry 4.0: Literature review and research opportunities in sustainable pharmaceutical supply chains. Process. Saf. Environ. Prot..

[16] De Lima F.R.P., Da Silva A.L., Filho M.G., Dias E.M. (2018). Systematic review: Resilience enablers to combat counterfeit medicines. Supply Chain Manag. Int. J..

[17] Da Silva R.B., De Mattos C.A. (2019). Critical Success Factors of a Drug Traceability System for Creating Value in a Pharmaceutical Supply Chain (PSC). Int. J. Environ. Res. Public Health.

[18] Bottoni P., Caroli S. (2019). Fake pharmaceuticals: A review of current analytical approaches. Microchem. J..

[19] Bolla A.S., Patel A.R., Priefer R. (2020). The silent development of counterfeit medications in developing countries—A systematic review of detection technologies. Int. J. Pharm..

[20] Yao W., Chu C.H., Li Z. The use of RFID in healthcare: Benefits and barriers. Proceedings of the 2010 IEEE International Conference on RFID-Technology and Applications.

[21] Tseng J.-H., Liao Y.-C., Chong B., Liao S.-W. (2018). Governance on the Drug Supply Chain via Gcoin Blockchain. Int. J. Environ. Res. Public Health.

[22] World Health Organization (1999). Counterfeit Drugs: Guidelines for the Development of Measures to Combat Counterfeit Drugs (No. WHO/EDM/QSM/99.1).

[23] Modgil S., Singh R.K., Hannibal C. (2021). Artificial intelligence for supply chain resilience: Learning from COVID-19. Int. J. Logist. Manag..

[24] Burns W. (2006). WHO launches taskforce to fight counterfeit drugs. Bull. World Health Organ..

[25] Bryatov S.R., Borodinov A.A. Blockchain technology in the pharmaceutical supply chain: Researching a business model based on Hyperledger Fabric. Proceedings of the International Conference on Information Technology and Nanotechnology (ITNT).

[26] Abbas K., Afaq M., Khan T.A., Song W.-C. (2020). A Blockchain and Machine Learning-Based Drug Supply Chain Management and Recommendation System for Smart Pharmaceutical Industry. Electronics.

[27] PSI (2019). Number of Incidents CY 2015–CY 2019 [Figure 8]. https://www.psi-inc.org/pharma-crime/incident-trends.

[28] Khan S., Haleem A., Khan M.I., Abidi M.H., Al-Ahmari A. (2018). Implementing Traceability Systems in Specific Supply Chain Management (SCM) through Critical Success Factors (CSFs). Sustainability.

[29] King B., Zhang X. Securing the pharmaceutical supply chain using RFID. Proceedings of the 2007 International Conference on Multimedia and Ubiquitous Engineering (MUE’07).

[30] Theyel G. Biomedical value chain traceability for innovation. Proceedings of the 2017 IEEE Technology & Engineering Management Conference (TEMSCON).

[31] Cohen D.F., Kirshner J. (2020). 6. The Cult of Energy Insecurity and Great Power Rivalry across the Pacific. The Nexus of Economics, Security, and International Relations in East Asia.

[32] Barchetti U., Bucciero A., De Blasi M., Mainetti L., Patrono L. RFID, EPC and B2B convergence towards an item-level traceability in the pharmaceutical supply chain. Proceedings of the 2010 IEEE International Conference on RFID-Technology and Applications.

[33] Salim M., Widodo R., Noordin M. (2021). Proof-of-Concept of Detection of Counterfeit Medicine through Polymeric Materials Analysis of Plastics Packaging. Polymers.

[34] Modgil S., Sonwaney V. (2019). Planning the application of blockchain technology in identification of counterfeit products: Sectorial prioritization. IFAC-PapersOnLine.

[35] Haji M., Kerbache L., Muhammad M., Al-Ansari T. (2020). Roles of Technology in Improving Perishable Food Supply Chains. Logistics.

[36] Jamil F., Ahmad S., Iqbal N., Kim D.-H. (2020). Towards a Remote Monitoring of Patient Vital Signs Based on IoT-Based Blockchain Integrity Management Platforms in Smart Hospitals. Sensors.

[37] Iqbal N., Jamil F., Ahmad S., Kim D. (2021). A Novel Blockchain-Based Integrity and Reliable Veterinary Clinic Information Management System Using Predictive Analytics for Provisioning of Quality Health Services. IEEE Access.

[38] Shahbazi Z., Byun Y.-C. (2020). A Procedure for Tracing Supply Chains for Perishable Food Based on Blockchain, Machine Learning and Fuzzy Logic. Electronics.

[39] Glover D.G., Hermans J. (2017). Improving the traceability of the clinical trial supply chain. Appl. Clin. Trials.

[40] Deisingh A.K. (2005). Pharmaceutical counterfeiting. Analyst.

[41] Woods J., Iyengar-Emens R. (2019). Blockchain to secure a more personalized pharma: Digital ledgers can detect fake drugs, enable virtual trials, and advance personalized medicine. Genet. Eng. Biotechnol. News.

[42] Schöner M.M., Kourouklis D., Sandner P., Gonzalez E., Förster J. (2017). Blockchain Technology in the Pharmaceutical Industry.

[43] Huang Y., Wu J., Long C. Drugledger: A practical blockchain system for drug Traceability and regulation. Proceedings of the 2018 IEEE International Conference on Internet of Things (iThings) and IEEE Green Computing and Communications (GreenCom) and IEEE Cyber, Physical and Social Computing (CPSCom) and IEEE Smart Data (SmartData).

[44] Kumar A., Choudhary D., Raju M.S., Chaudhary D.K., Sagar R.K. Combating counterfeit drugs: A quantitative analysis on cracking down the fake drug industry by using blockchain technology. Proceedings of the 2019 9th International Conference on Cloud Computing, Data Science & Engineering (Confluence).

[45] Kumar R., Tripathi R. Traceability of counterfeit medicine supply chain through Blockchain. Proceedings of the 2019 11th International Conference on Communication Systems & Networks (COMSNETS).

[46] Shahbazi Z., Byun Y.-C. (2021). Integration of Blockchain, IoT and Machine Learning for Multistage Quality Control and Enhancing Security in Smart Manufacturing. Sensors.

[47] Gautam C.S., Utreja A., Singal G.L. (2009). Spurious and counterfeit drugs: A growing industry in the developing world. Postgrad. Med. J..

[48] Klein K., Stolk P. (2018). Challenges and Opportunities for the Traceability of (Biological) Medicinal Products. Drug Saf..

[49] Sharifian R., Ebrahimi S., Bastani P. (2015). How radio frequency identification improves pharmaceutical industry: A comprehensive review literature. J. Pharm. Care.

[50] Nilsson E., Nilsson B., Järpe E. A pharmaceutical anti-counterfeiting method using time controlled numeric tokens. Proceedings of the 2011 IEEE International Conference on RFID-Technologies and Applications.

[51] Ng E.H., Nepal B., Schott E., Keathley H. Internet of things: Opportunities and applications in pharmaceutical manufacturing and logistics. Proceedings of the American Society for Engineering Management 2018.

[52] Pérez M.M., González G.V., Dafonte C. (2016). Safety and Traceability in Patient Healthcare through the Integration of RFID Technology for Intravenous Mixtures in the Prescription-Validation-Elaboration-Dispensation-Administration Circuit to Day Hospital Patients. Sensors.

[53] Del Carmen León-Araujo M., Gómez-Inhiesto E., Acaiturri-Ayesta M.T. (2019). Implementation and evaluation of a RFID smart cabinet to improve traceability and the efficient consumption of high cost medical supplies in a large hospital. J. Med. Syst..

[54] Thatcher C., Acharya S. Pharmaceutical uses of blockchain technology. Proceedings of the 2018 IEEE International Conference on Advanced Networks and Telecommunications Systems (ANTS).

[55] Jamil F., Hang L., Kim K., Kim D. (2019). A Novel Medical Blockchain Model for Drug Supply Chain Integrity Management in a Smart Hospital. Electronics.

[56] Imran, Iqbal N., Ahmad S., Kim D. (2021). Health Monitoring System for Elderly Patients Using Intelligent Task Mapping Mechanism in Closed Loop Healthcare Environment. Symmetry.

[57] Iqbal N., Imran, Ahmad S., Ahmad R., Kim D.-H. (2021). A Scheduling Mechanism Based on Optimization Using IoT-Tasks Orchestration for Efficient Patient Health Monitoring. Sensors.

[58] Breen L. (2008). A Preliminary Examination of Risk in the Pharmaceutical Supply Chain (PSC) in the National Health Service (NHS). J. Serv. Sci. Manag..

[59] Tremblay M. (2013). Medicines counterfeiting is a complex problem: A review of key challenges across the supply chain. Curr. Drug Saf..

[60] Punniyamoorthy M., Thamaraiselvan N., Manikandan L. (2013). Assessment of supply chain risk: Scale development and validation. Benchmarking Int. J..

[61] Narayana S.A., Pati R.K., Vrat P. (2012). Research on management issues in the pharmaceutical industry: A literature review. Int. J. Pharm. Health Mark..

[62] Lee K., Kassab Y., Taha N., Zainal Z. (2020). Factors Impacting Pharmaceutical Prices and Affordability: Narrative Review. Pharmacy.

[63] Li M., Du Y., Wang Q., Sun C., Ling X., Yu B., Tu J., Xiong Y. (2016). Risk assessment of supply chain for pharmaceutical excipients with AHP-fuzzy comprehensive evaluation. Drug Dev. Ind. Pharm..

[64] Soon J.M., Manning L. (2019). Developing anti-counterfeiting measures: The role of smart packaging. Food Res. Int..

[65] Singh R., Kumar R., Kumar P. (2016). Strategic issues in pharmaceutical supply chains: A review. Int. J. Pharm. Health Mark..

[66] Mäkinen M.M., Rautava P.T., Forsström J.J. (2005). Do online pharmacies fit European internal markets?. Health Policy.

[67] Gray N. (2011). The evolution of online pharmacies. Self Care J..

[68] Leontiadis N., Moore T., Christin N. Pick your poison: Pricing and inventories at unlicensed online pharmacies. Proceedings of the Fourteenth ACM Conference on Electronic Commerce.

[69] Shaikh Z.A., Buzdar M.H., Pahore M.R., Mirani I.H., Noor M. (2019). The Emergent Business of Internet Pharmacies: Convenience, Risks, Regulatory Policies and Future. J. Pharm. Res. Int..

[70] Stank T.P., Keller S.B., Daugherty P.J. (2001). Supply chain collaboration and logistical service performance. J. Bus. Logist..

[71] Barratt M. (2004). Understanding the meaning of collaboration in the supply chain. Supply Chain Manag. Int. J..

[72] Min S., Roath A.S., Daugherty P.J., Genchev S.E., Chen H., Arndt A.D., Richey R.G. (2005). Supply chain collaboration: What’s happening?. Int. J. Logist. Manag..

[73] Matopoulos A., Vlachopoulou M., Manthou V., Manos B. (2007). A conceptual framework for supply chain collaboration: Empirical evidence from the agri-food industry. Supply Chain Manag. Int. J..

[74] Fawcett S.E., Magnan G.M., McCarter M.W. (2008). Benefits, barriers, and bridges to effective supply chain management. Supply Chain Manag. Int. J..

[75] De Vries J., Huijsman R. (2011). Supply chain management in health services: An overview. Supply Chain Manag. Int. J..

[76] Karlsen K., Sørensen C., Forås F., Olsen P. (2011). Critical criteria when implementing electronic chain traceability in a fish supply chain. Food Control.

[77] Papert M., Rimpler P., Pflaum A. (2016). Enhancing supply chain visibility in a pharmaceutical supply chain. Int. J. Phys. Distrib. Logist. Manag..

[78] Tian F. A supply chain traceability system for food safety based on HACCP, blockchain & Internet of Things. Proceedings of the 2017 International Conference on Service Systems and Service Management.

[79] Fernando E. Success factor of implementation blockchain technology in pharmaceutical industry: A literature review. Proceedings of the 2019 6th International Conference on Information Technology, Computer and Electrical Engineering (ICITACEE).

[80] Sodhi M.S., Tang C.S. (2019). Research Opportunities in Supply Chain Transparency. Prod. Oper. Manag..

[81] Hastig G.M., Sodhi M.S. (2019). Blockchain for Supply Chain Traceability: Business Requirements and Critical Success Factors. Prod. Oper. Manag..

[82] Rogerson M., Parry G.C. (2020). Blockchain: Case studies in food supply chain visibility. Supply Chain Manag. Int. J..

[83] Subramanian L. (2020). Enabling health supply chains for improved well-being. Supply Chain Forum: Int. J..

[84] Khanuja A., Jain R.K. (2019). Supply chain integration: A review of enablers, dimensions and performance. Benchmarking Int. J..

[85] Cagliano A.C., Mangano G., Rafele C. (2021). Determinants of digital technology adoption in supply chain. An exploratory analysis. Supply Chain Forum: Int. J..

[86] Huang Y., Han W., Macbeth D.K. (2020). The complexity of collaboration in supply chain networks. Supply Chain Manag. Int. J..

[87] Zekhnini K., Cherrafi A., Bouhaddou I., Benghabrit Y., Garza-Reyes J.A. (2020). Supply chain management 4.0: A literature review and research framework. Benchmarking Int. J..

[88] Razak G.M., Hendry L.C., Stevenson M. (2021). Supply chain traceability: A review of the benefits and its relationship with supply chain resilience. Prod. Plan. Control.

[89] Sunny J., Undralla N., Pillai V.M. (2020). Supply chain transparency through blockchain-based traceability: An overview with demonstration. Comput. Ind. Eng..

[90] Akhtar M.M., Rizvi D.R. (2021). Traceability and detection of counterfeit medicines in pharmaceutical supply chain using blockchain-based architectures. Sustainable and Energy Efficient Computing Paradigms for Society.

[91] Soundarya K., Pandey P., Dhanalakshmi R. (2018). A Counterfeit Solution for Pharma Supply Chain. EAI Endorsed Trans. Cloud Syst..

